# The impact of a communal multidisciplinary tumour board on medical education

**DOI:** 10.3332/ecancer.2024.1788

**Published:** 2024-10-11

**Authors:** Oluwaseyi K Idowu, Adewumi O Alabi, Ibijoke A Idowu, Opeyemi I Olusunmade, Bright A Igbinoba, Abdulwahab Ajani, Mohammed Y M Habeebu, Jane U Igwilo, Kolawole O Aramide, Eyitayo O Alabi, Fatimah B Abdulkareem, Samuel U Eyesan, Suleiman O Giwa

**Affiliations:** 1 Department of Oncology, National Orthopaedic Hospital, Igbobi, Lagos 2008, Nigeria; 2 Department of Radiation Biology, Radiotherapy & Radiodiagnosis, College of Medicine, University of Lagos, Lagos 12003, Nigeria; 3 Faculty of Engineering and Technology, Liverpool John Moores University, Liverpool L3 5UX, UK; 4 Department of Radiodiagnosis, National Orthopaedic Hospital, Igbobi, Lagos 2008, Nigeria; 5 Department of Pathology, National Orthopaedic Hospital, Igbobi, Lagos 2008, Nigeria; 6 Department of Trauma & Orthopaedics, Lagos University Teaching Hospital, Lagos 12003, Nigeria; 7 Department of Anatomic & Molecular Pathology, College of Medicine, University of Lagos, Lagos 12003, Nigeria

**Keywords:** multidisciplinary team, multidisciplinary team meetings, musculoskeletal tumours, medical education, low middle-income countries

## Abstract

**Introduction:**

Numerous challenges hinder the development of multidisciplinary medical education in a resource-constrained environment. Communal tumour boards built through networking could be a suitable model for the effective management of diseases and enhancement of medical education. This study evaluated the impact of an integrated care pathway for patients with musculoskeletal tumours via multi-institutional networking in a metropolis.

**Methodology:**

Musculoskeletal tumours managed in different institutions in a large metropolis were included for discussion at monthly meetings, under the aegis of the Lagos Musculoskeletal Oncology Network (LAMON). The cases discussed were collated and presented by designated senior residents. The meetings ensured adherence to agreed national and international guidelines in the management of musculoskeletal tumours. Decisions about the treatment modalities were planned at the meetings. The impact on medical residency training was evaluated using the achievement of significant milestones by the residents supported by the network.

**Results:**

The tumour board network included health professionals from various specialist hospitals in the metropolis. Within the decade (2013–2022), 1,272 patients were reviewed of which 968 patients had definitive histological diagnoses. There was an improvement in limb salvage rate and disease outcome. The tumour board supported significant milestones in graduate medical training, including the completion of 4 residents’ fellowship dissertations, 22 conference presentations by residents, the publication of 12 articles by residents and the completion of an orthopaedic oncology subspecialty fellowship by 9 orthopaedic surgeons.

**Conclusion:**

The tumour board/network improved the outcome of musculoskeletal tumours over the study period. The network improved the education of medical doctors and increased the capacity for training newer instructors in a resource-limited environment. Perhaps with appropriate social and corporate support, communal tumour boards like LAMON may translate into a good model for multidisciplinary care of diseases and capacity building in resource-limited settings.

## Introduction

Multidisciplinary healthcare teams work by collaborating to optimise patient care. The connections between the multispecialties contribute greatly to optimal care of diseases like musculoskeletal tumours [1]. Previous surveys amongst medical teachers suggest that educational quality is enhanced by the efficiency of multidisciplinary teams (MDTs) involved in curriculum development [2]. Multidisciplinary team meetings (MDTm) enhance graduate medical education by providing a better understanding of specific disease conditions. This invariably contributes to the attainment of significant milestones during and after graduating medical training [1–3].

Numerous challenges hinder the treatment of musculoskeletal tumours in resource-constrained environment, such as sub-Saharan Africa. A prospective study of bone and soft-tissue tumours revealed challenges with appropriate imaging, procurement of chemotherapy and limb-sparing surgeries [4]. Funding constraints (due to out-of -pocket payments), inadequate specialist personnel, cultural and religious factors contribute significantly in delayed diagnosis and treatment of cancers especially musculoskeletal cancers [4, 5].

Previous research conducted in the same metropolis as this study showed the most prevalent histological diagnoses of primary bone tumours included osteosarcoma and soft tissue sarcoma [6]. Adigun *et al* [5] evaluated the presentation of soft tissue sarcoma and reported that the peak age incidence was between the third and sixth decades of life with a slight male preponderance. Fibrosarcoma was the commonest soft tissue sarcoma followed by malignant fibrous histiocytoma, liposarcoma and rhabdomyosarcoma [5]. The patients presented with delayed and advanced stages of diseases, and limb salvage was only possible in 52.6% of these patients [5]. Considering the dismal survival rate from these previous studies, the authors suggested that optimal results of treatment require multidisciplinary interaction [5, 7].

In the last decade, multidisciplinary tumour boards or networks have emerged in Nigeria to mitigate these previous challenges in managing malignancies. Hitherto, most of the treatment was provided by individual specialists within the same institution, hence there was limited collaboration amongst specialties in nearby institutions to enhance robust cancer care. Infact until recently, MDTm was not a mandatory requirement for cancer care in public institutions in Nigeria, leading to a paucity of data on existing MDTs [8].

There is robust evidence that MDTm provides adequate communication between all providers and disciplines involved in treatment [9]. The adoption of an innovative care framework for chronic conditions such as musculoskeletal cancers should be flexible, and tailored to particular health systems, such that it is sustainable within the local resources and demands [10].

Studies addressing the impact of MDTs in different regions and countries are sparse. There is a great disparity in the structure, organisation and frequency of the MDTm in different countries and regions around the world. In a large international survey among cancer specialists, 65% of the respondents from Eastern Europe, 63% from Western Europe, 35% from Asia and 25% from South America declared that MDTm was a mandatory part of cancer care in their country [11]. Ninety percent of the respondents from Europe stated their MDT met weekly, compared with only 50% of the respondents from Asia [11].

The main drawback of the institution of MDTm are, potential breaches of patient confidentiality (patients’ information is circulated widely), medico-legal implications of MDT decisions and culpability. Also, MDTm in most wealthy countries involve a significant salary burden on the healthcare system, since specialists are paid for their participation at the scheduled meetings. In most low and middle-income countries (LMICs) there is no protected time for clinicians to attend the MDTm [12]. Nevertheless, MDTs may be particularly more important in LMICs, where patients present in more advanced stages of cancer require multimodality treatment [13]. There is evidently no perfect healthcare system, even the wealthiest countries are compelled to stringently manage healthcare resources [14]. MDTm has been adjudged cost effective in a study comparing the cost per quality‐adjusted life year amongst two cohorts of cancer patients [14]. The patients were grouped into those who had MDT input and those who were not reviewed at an MDT meeting [14]. The cost per quality-adjusted life year was compared to pre- and post-MDT implementation [14].

Disease-specific communal boards through networking (where physician volunteer their time) could be a suitable option for the effective management of specific chronic diseases and the enhancement of medical education. This study examined the clinical impact of an integrated care pathway for patients with musculoskeletal tumours via multi-institutional networking in a metropolis. It also definitively evaluated the educational impact on surgical resident trainees.

## Methods

Musculoskeletal tumor patients treated at various institutions in a large metropolis are discussed monthly at the MDTm, organised by the Lagos Musculoskeletal Oncology Network (LAMON) established in 2013. The institutions included; National Orthopaedic Hospital Lagos (NOHIL), Lagos University Teaching Hospital Lagos, Lagos State University Hospital Lagos and State General Hospitals Lagos. The meetings ensured adherence as much as possible to agreed national and international guidelines in the management of musculoskeletal tumours. The cases discussed were collated and presented by designated senior residents.

All MDTm cases to be discussed were anonymised and shared with participants for review via emails or secured group chat. It was the prerogative of the presenting department and attending clinicians to decide which cases would be presented, but many of the cases were newly diagnosed musculoskeletal neoplasms. Before the meeting, pathology slides and diagnostic images were reviewed by the pathologists and the radiologists respectively. These were also projected and reviewed during the MDTm and inputs were sought from all participants.

The study period was from January 2013 to December 2022. In the initial phase (year 2013–2019), the meetings were conducted in-person (physical meetings) at a single venue (NOHIL). However, with the advent of the COVID restrictions the meetings were conducted virtually via the Zoom app (Zoom Video Communications, Inc. San Jose, US) from the year 2020–2022. The residents in different specialties presented the cases, and attendings/consultants contributed their opinions on the cases. The agenda was structured into radiology, histopathology and other business sessions. Decisions about surgery, chemotherapy, radiotherapy and timing of the modalities, were planned at the meetings. Research protocols in musculoskeletal oncology designed by residents were also evaluated at the MDT meetings.

The secretary of the meetings recorded all recommendations in pre-designed MDT registry/database. The meetings were supported by a charity group dedicated to the care of patients with musculoskeletal tumours, Musculoskeletal Oncology Support Foundation (MONSUF).

The network benefited from regular contributions from four pathologists, three radiation oncologists, five radiologists, three plastic surgeons, nine orthopaedic surgeons and two general surgeons working in various specialist hospitals within the metropolis. Social workers, physiotherapists, oncology specialist nurses and pharmacists also attended regularly.

The charity group MONSUF, provided financial support for indigent patients reviewed within the network. MONSUF also provided support for the residents’ research proposals.

The number of cases presented per meeting, number of specialists in attendance, reasons for case presentation and recommendation of the MDTm were recorded.

The impact of LAMON MDTm on residency training was evaluated using the attainment of some significant milestones, i.e, number of fellowship dissertations completed in musculoskeletal oncology, number of related articles published by residents as coauthors, number of related papers presented by residents at conferences and the number of residents choosing musculoskeletal oncology subspecialty for post-fellowship training.

This study was approved by the NOHIL Ethics Committee. For confidentiality purposes, each case was coded by a unique identifier. Data were recorded on Microsoft Excel and then to IBM SPSS, version 27.0 for Windows (IBM Corp) for statistical analysis. Descriptive statistics were reported. For continuous variables, mean or median and interquartile ranges were reported. For categorical variables, frequencies and percentages were reported.

## Results

**Figure 1.**
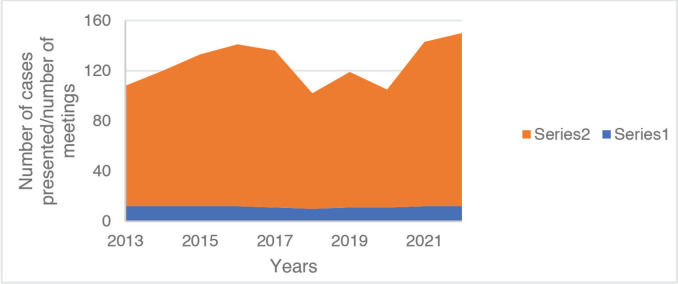
The MDT meetings and associated cases presented per year.

In the study period (January 2013–December 2022) 1,142 patients were reviewed of which 968 patients (85%) had definitive histological diagnoses (Table 1 and Figure 1). The age range of the patients was 2–95 years. There were 639 males (56%) and 503 females (44%). The tumour was located on the extremity in 82%(936) of cases and centrally in 18% (206). The common histological diagnoses include metastatic bone disease, osteosarcoma and soft tissue sarcoma.

**Figure 2.**
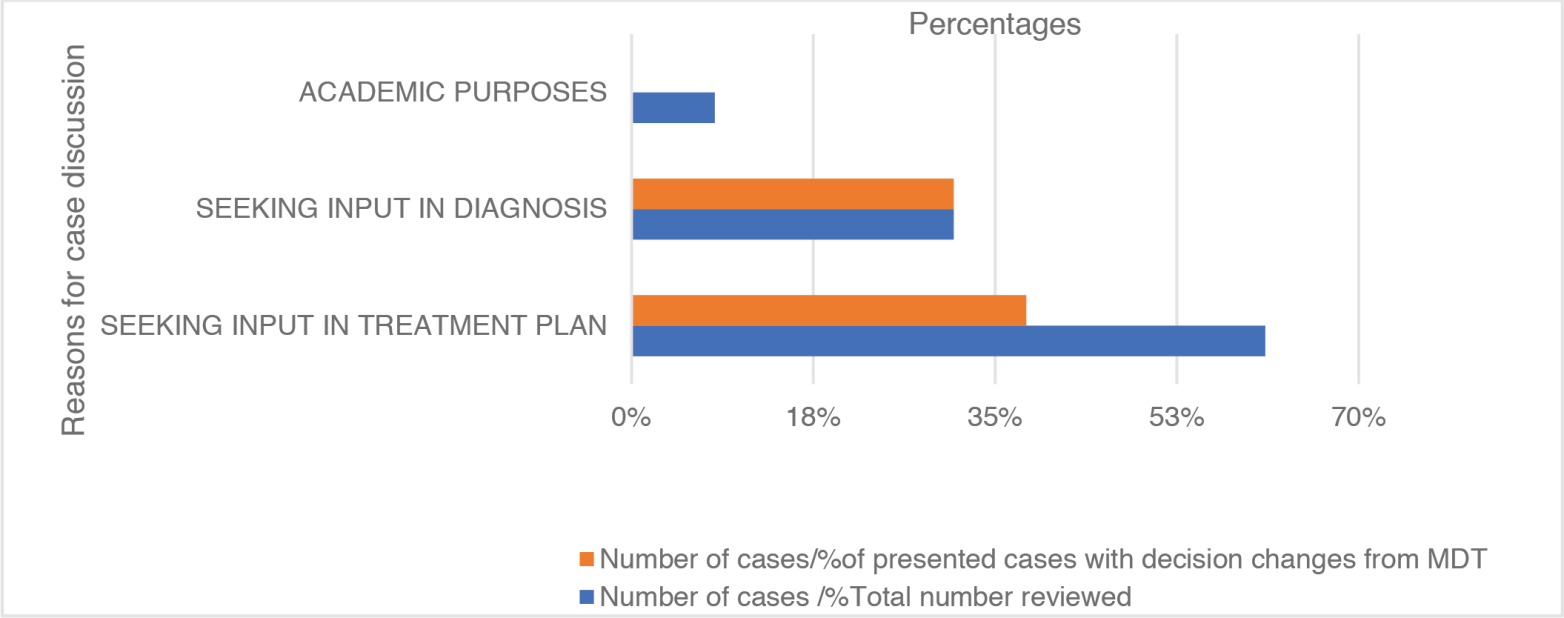
Overview of the reasons for discussion at MDT.

During the MDT discussion, input was sought for treatment plans for 697 (61%) patients and diagnoses for 354 (31%) patients. While the cases were presented for educational purposes in 91(8%) patients reviewed. Following review by the network, the MDT input led to changes in the initial treatment plan by the attending unit in 264 (38%)patients. The MDT input led to changes in initial diagnostic strategies in 110 (31%) patients (Table 2 and Figure 2).

**Figure 3.**
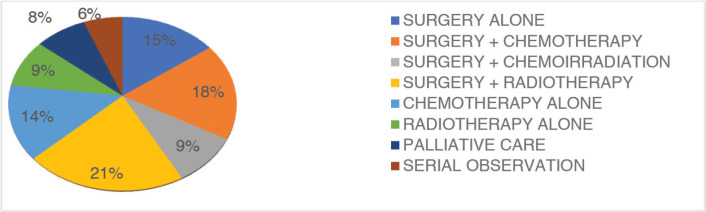
Prescriptive recommendation post MDT.

The common recommendations of the MDT include surgery + radiotherapy(21%), surgery + chemotherapy(18%) and surgery alone (15%) (Table 3 and Figure 3). Other recommendations included, chemotherapy alone, radiotherapy alone, palliative care and serial observation.

**Figure 4.**
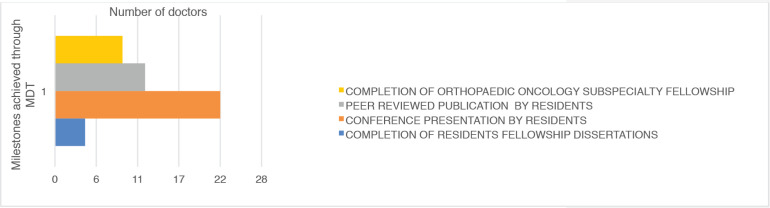
Milestones achieved through MDT.

Amputation was the surgical treatment offered in 394 patients (34.5%) whereas, 748 patients (65.5%) required limb salvage procedures and/or adjuvant treatment. There was a local recurrence of the disease in 94 patients (8.3%) (Table 4 and Figure 4).

Moreover, the tumour board supported significant milestones in graduate medical training within the study period through the MDTm. The network provided conception and guidance for different educational projects for resident doctors. These included, the completion of 4 resident fellowship dissertations, 22 conference presentations by residents, the publication of 12 articles by residents and the completion of Orthopaedic Oncology subspecialty fellowship by 9 Orthopaedic surgeons.

## Discussion

This study sought to evaluate the impact of a communal tumour MDT on patient care and on graduate medical education. The network was appropriately constituted, with support from pathology, surgery, radio-diagnosis and radio-oncology specialties. The objective of this MDT was to improve case recognition, time to diagnosis and overall outcome in musculoskeletal tumours by harmonisation of decisions on patient care. The hypothesis is that this should translate to improvement in doctors’ training in the appropriate management of site-specific diseases like musculoskeletal neoplasm.

The study highlighted that the majority of the patients (85%) in the study period had definite histological diagnosis. This is a significant improvement when compared with a previous study in the same region before the institution of the LAMON MDT. The most common histological diagnoses were metastatic bone disease, osteosarcoma and soft tissue sarcoma, which showed a similar presentation pattern to previous studies done in the same institution [5, 7].

The MDTm influenced the treatment plan in 264 (38%) patients and influenced diagnostic strategies in 110 (31%) patients. The common recommendations of the MDTm in our study were, surgery + radiotherapy (21%), surgery + chemotherapy (18%) and surgery alone (15%). This contributed to the limb salvage rate of 65.5% amongst patients discussed at the MDTm. The results represent a significant improvement in the approach to treatment and outcome of musculoskeletal tumours when compared to previous studies before the institution of the MDT in the same metropolis [4, 7].

The case documentation and development of a database improved, and a better outcome was achieved through the resourceful use of diagnostic (radiologic/laboratory) and treatment (chemotherapy/radiotherapy) modalities. Previous reports have identified enormous challenges in obtaining investigations and administering therapies for patients with musculoskeletal malignancies in a tertiary musculoskeletal tumour service in the same metropolis [4, 7, 13, 15]. The development of the database contributed to the medical education and research potential.

The MDTm enabled better surgical planning, access to adjuvant therapy and follow up of patients within the communal network. The advent of mobile phone applications facilitated better communication within the communal network, and with patients and their relatives. Resourcefulness via MDT networks is of particular importance in LMIC where the health insurance system is poorly developed and patients have to pay out of pocket at the point of service.

Previous authors demonstrated a relationship between scholarly productivity based on journal publication and clinical performance during and after residency training [16]. There is evidence that residents who invest substantial efforts in research improve their abilities to learn medicine and care for patients [16–19].

The tumour board supported significant milestones in graduate medical training within the study period. Hitherto, musculoskeletal oncology was an unpopular area of study amongst residents applying for postgraduate examinations. The MDT (and the charity group) encouraged the conception and supervision of four resident fellowship dissertations. These studies were well supported and supervised by specialists within the LAMON MDT. The themes of the dissertations include; better diagnostic methods for bone tumours with soft tissue components, reinforcement of specificity of the magnetic resonance imaging in the diagnosis of musculoskeletal tumours, and the role of biomarkers in earlier diagnosis of musculoskeletal tumours [20–29].

This MDT improved the research skills of the resident doctors by supporting and supervising 22 conference paper presentations and the publication of 12 full articles by resident doctors. This culminated in the initiation of the first musculoskeletal oncology specialty fellowship program in the subregion. Four residents have completed this musculoskeletal oncology subspecialty fellowship.

Moreover, Walraven *et al* [30] in a survey among a group of Dutch residents and specialists identified MDT as a viable medium for preparing residents for their future role as specialists. The authors suggested better guidance by supervisors to support residents during the meetings and clearer regulations to enhance active participation [30]. Interviewees in this study indicated a need for additional training (e.g., simulations) for residents, especially to enhance behavioural and communication skills within the MDT fora (e.g., being well prepared, sitting in the inner circle, having assigned responsibilities).

Furthermore, in a survey among surgical resident doctors within two regions in the UK, Trivedi [31] reported that 71.42% of respondents attended MDT at least once a week. This study also reported that 75.9% of participants indicated the importance of attendance to MDT meetings with any level of involvement. Resident doctors also felt included in the team by attending MDT meetings and the meetings were considered important for learning all domains of non-technical skills for surgeons’ taxonomy.

Most of the respondents regarded MDTm as a valuable tool for learning non-technical skills for surgeons on Miller’s pyramid of learning. The results indicate consistently positive views from trainees about the educational value of MDT meetings in general and for non-technical skills. Resident doctors’ participation in case-preparation, presentation and discussion is encouraged.

While the meetings were not built into the contract schedules of individual consultants within the network, the meetings were sustained by the conviction of adequate quality assurance it provided for the healthcare community. This network enhanced clinical documentation and provided a database for patients with musculoskeletal tumours.

## Conclusion and recommendation

The tumour board/network improved the treatment approach, and outcome of musculoskeletal tumours over the study period. The network improved the education of medical doctors and increased the capacity for training newer instructors in a resource-limited environment. There is a need for further studies on the value and problems of communal MDTs in other site-specific disease conditions. Perhaps with appropriate social and corporate support, communal tumour boards like LAMON MDT may translate into model for multidisciplinary cancer care and capacity building in LMIC.

## Conflicts of interest

The authors report no conflicts of interest in this work as the research received no sponsorship or financial support from any individual or organisation.

## Funding

No funding was obtained.

## Figures and Tables

**Table 1.** MDT meetings and cases presented per year.

Year	Number of meetings	Number of cases presented
2013	12	96
2014	12	108
2015	12	121
2016	12	129
2017	11	125
2018	10	92
2019	11	108
2020	11	94
2021	12	131
2022	12	138
Total	115	1,142

**Table 2.** Reasons for discussion at MDT.

Reason for case discussion	Number of cases/percentage of total number reviewed	Number / percentage of presented cases with decision changes from MDT
Seeking input in treatment plan	697 (61%)	264 (38%)
Seeking input in diagnosis	354 (31%)	110 (31%)
Academic purposes	91 (8%)	NIL

**Table 3.** Recommendation post MDT.

Recommendation	Number/Percentage
Surgery alone	171 (15%)
Surgery + Chemotherapy	206 (18%)
Surgery + Chemoradiation	103 (9%)
Surgery + Radiotherapy	240 (21%)
Chemotherapy alone	159 (14%)
Radiotherapy alone	103 (9%)
Palliative care	91 (8%)
Serial observation	69 (6%)

**Table 4.** MDT supported significant milestones.

Milestone	Number of doctors
Completion of residents fellowship dissertations	4
Conference presentation by residents	22
Peer reviewed publication by residents	12
Completion of orthopaedic oncology subspecialty fellowship	9
